# Supplementary material for: UniVector: Unified vector extraction via instance-geometry interaction
Source: Fundam Res. 2026 May 13;6(5):3319–29. doi: 10.1016/j.fmre.2026.03.025 (PMC13644518; doi:10.1016/j.fmre.2026.03.025)
Supplement: Supplementary file 1 [file mmc1.pdf]

Supplementary Materials
UniVector: Unified Vector Extraction via Instance-Geometry Interaction

Abstract

In the supplementary materials, we provide a detailed explanation of the motivation behind the instance–geometry interaction, a comprehensive introduction to the Multi-Vector dataset, the specific experimental setup, and additional experimental data and visualizations not included in the main text.

1. Supplementary Materials

1.1. The Motivation of Instance–geometry Interaction

Previous methods [1], [2], [3] primarily adopt a cascaded approach, modeling vector instances and geometric attributes separately while overlooking the information gap between these levels. As illustrated in Fig. 2(a), vectors of different structures inherently contain both instance-level and geometric-level attributes [4], [5]. Instance-level attributes capture global characteristics, such as semantic categories and structural characteristics (connectivity and closeness), whereas geometric-level attributes explicitly define vector shapes through point coordinates and connection relationships. For the representation of vectors, we observe that relying solely on instance attributes struggles to accurately capture the geometric shape of targets, while using only geometric attributes fails to ensure correct instance topology. When we utilize a joint representation of both attributes, we can accurately describe diverse structures (see Fig. 2(b)). Therefore, previous cascaded approaches (Fig. **??**(a) and (b)) inherently constrain the model's capacity to represent and learn diverse vector structures. To address this, we model the interaction between instance and geometric attributes to bridge the information gap and achieve cross-level complementarity. This allows instance attributes to provide global structural priors for maintaining correct topology and spatial

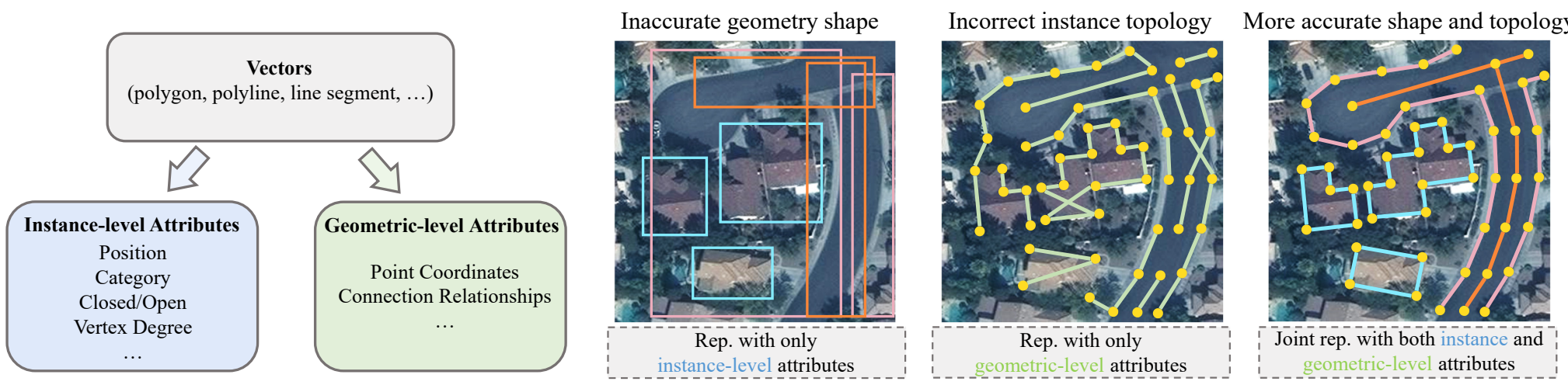

(a) Instance and geometry-level attributes

(b) Comparison of different representation in multi-structure VE

Figure 1: Illustration of our motivation. (a) Different structures possess both instance-level and geometry-level attributes [4], [5]. (b) Relying solely on instance attributes leads to inaccurate geometric shapes, while focusing only on geometric attributes results in incorrect instance topology. A joint representation of instance and geometric attributes enables more accurate shape and topology reconstruction in multi-structure VE.

coherence, while geometric attributes aid in distinguish the semantic and structural properties of instances more effectively (Fig. **??**(c)).

1.2. Multi-Vector Dataset

In this work, we constructed the first multi-structure vector extraction dataset (Multi-Vector), which encompasses multiple categories and structural types, filling the gap in the field of UVE for diverse structures (Table 1). The Multi-Vector dataset consists of 20,000 training images and 3,734 validation images, covering three semantic categories: buildings, road boundaries, and center lines. Notably, while existing vector extraction datasets focus on specific vector structures, UniVector includes three distinct vector structures: polygons, polylines, and line segments (see Fig. 2(b)). Building on the extensive building vector data from CrowdAI [6], we re-annotated road boundaries and center lines commonly used in vector maps [7], [8]. The geometric structures of these vectors are represented as directed point sequences, with annotations formatted in the widely adopted COCO [9] instance segmentation format. The dataset distribution is 70.6% buildings, 18.9% road boundaries, and 10.5% center lines. As shown in Fig. **??**(a), buildings are represented exclusively by polygons, while road boundaries and center lines are represented by polylines and line segments, respectively. This makes the Multi-Vector dataset more aligned with practical applications and presents greater challenges compared to previous datasets.

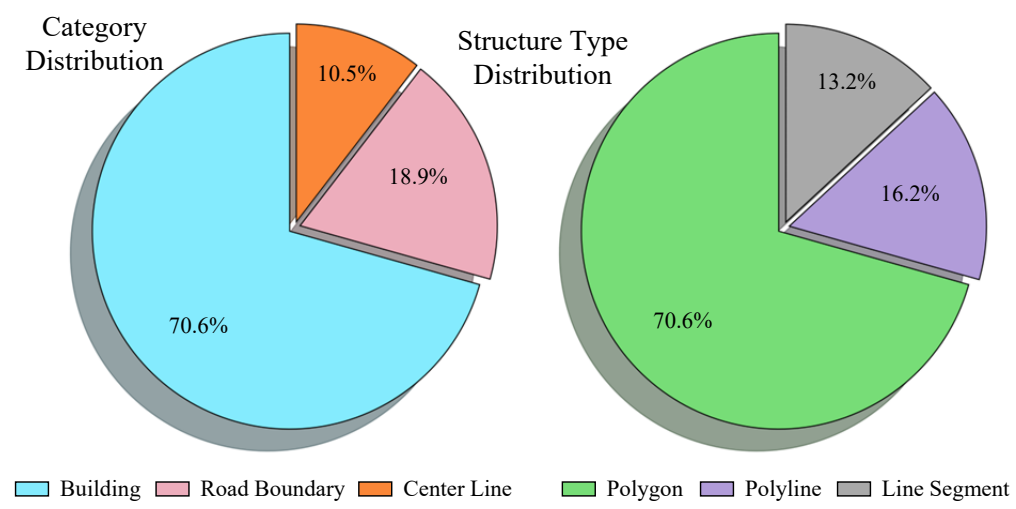

(a) Category and structure distribution of Multi-Vector

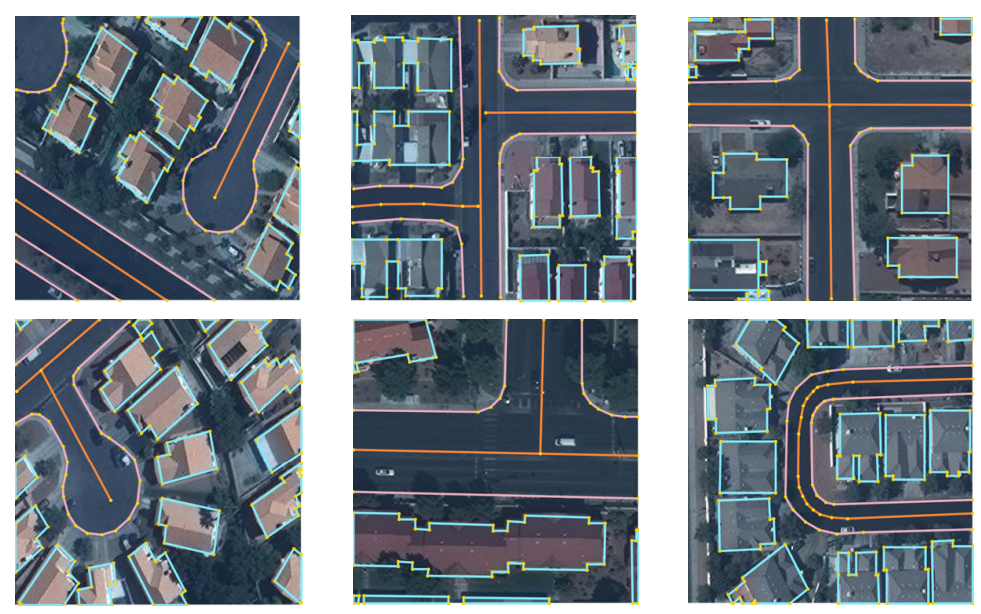

(b) Example images and annotations of Multi-Vector

Figure 2: Category and structure distribution of the Multi-Vector dataset and annotation samples.

1.3. Experiment Settings

For the Multi-Vector dataset, we set the number of vector instances per image to 50 and the number of points per vector to 40. ResNet50 [21] is used as the backbone network, unless otherwise specified. The optimizer is AdamW with a weight decay of 0.01, and the batch size is set to 6. All models are trained using 4 NVIDIA GeForce RTX 3090 GPUs. The default training schedule is set to 30 epochs, with the initial learning rate configured to 1×10^{-4}, decaying by a factor of 10 at the 27th epoch. In the dynamic shape constraints, the matching cost factors a and b are set to 5 and 2, respectively, while the vector shape supervision parameters α_1, α_2, and α_3 are set to 1, 10, and 1, respectively. The loss function consists of instance-level and geometric-level losses. Instance-level losses include classification loss, bounding box loss, and GIoU loss, with specific configurations consistent with Dino [22]. Geometric-level losses comprise point coordinate loss [23], point classification loss, and dynamic shape constraint. The corresponding loss weights are empirically set to 2.0, 1.0, and 1.0, respectively. For parameters specific to other datasets, please refer to the configuration file in the code

Table 1: Comparison of the Multi-Vector dataset with existing vector extraction datasets. The Multi-Vector dataset includes multiple semantic categories and vector structures simultaneously.

Dataset	Category	Vector Structure			Number	Instance Number
		Polygon	Polyline	Line Segment		
CrowdAI [6]	Building	✓			341,058	8.6/image
Inria [10]	Building	✓			360	11.7/image
Structured3D [11]	Room	✓			3,500	6.2/image
SceneCAD [12]	Room	✓			955	1.0/image
COCO [9]	80 Classes of Common Objects	✓			140,000	7.3/image
SBD [13]	20 Classes of Common Objects	✓			11,355	1.5/image
Topo-Boundary [14]	Road Boundary		✓		25,295	5.6/image
Tusimple [15]	Lane		✓		6,408	4.0/image
CULanes [16]	Lane		✓		133,235	4.0/image
LLAMAS [17]	Lane		✓		200,000	4.0/image
OpenLane [7]	14 Classes of Lanes		✓		100,042	7.0/image
Wireframe [18]	Wireframe			✓	5,462	74.2/image
York Urban [19]	Wireframe			✓	102	118.8/image
SEL [20]	Semantic Line			✓	1,715	1.63/image
NKL [15]	Semantic Line			✓	6,500	2.0/image
**Multi-Vector (Ours)**	Building, Road Boundary, Center Line	✓	✓	✓	23,734	12.1/image

Table 2: Comparison of training and inference times on the Multi-Vector dataset. * denotes the minimum sum and †denotes the maximum sum.

Method	Structure	Training	Inference
Specific-structure Vector Extraction			
HiSup [24]	polygon	0.11 s/image	0.13 s/image
PolyR-CNN [1]	polygon	0.24 s/image	0.15 s/image
RNGDet++ [25]	polyline / line segment	1.31 s/image	1.73 s/image
SAM-Road [26]	polyline / line segment	0.12 s/image	0.16 s/image
Multi-structure Vector Extraction			
Multi-model Casecad*	polygon, polyline, line segment	0.35 s/image	0.45 s/image
Multi-model Casecad†	polygon, polyline, line segment	2.86 s/image	3.59 s/image
**UniVector**	**polygon, polyline, line segment**	**0.14 s/image**	**0.17 s/image**

repository [1].

1.4. Comparison with State-of-the-Art Methods

1.4.1. Multi-structure Vector Extraction

Notably, UniVector enables the simultaneous extraction of multiple vector structures, eliminating the need for sequential extraction in practical applications. As shown in Table 2, compared to traditional cascaded multi-model approaches, UniVector demonstrates 2.5× faster training and 2.35× accelerated inference. When the cascaded system incorporates iterative inference models, these efficiency improvements can reach up to 20×.

1.4.2. Specific-structure Vector Extraction

Polygon Extraction. In Table 3, our method achieves superior performance in room-level accuracy, recall, and F1-score compared to RoomFormer.

[1] `https://github.com/yyyyll0ss/UniVector`

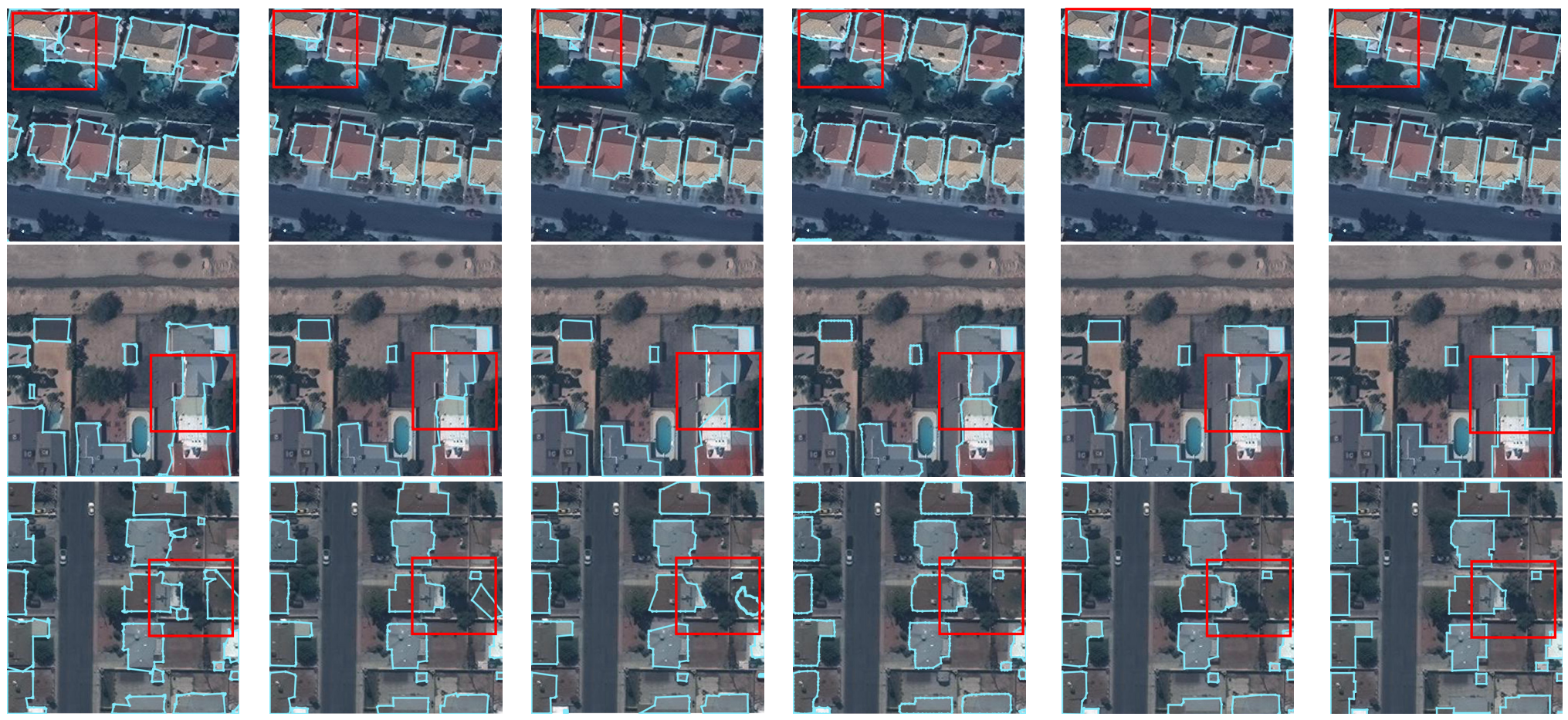

Figure 3: Qualitative comparison with other building extraction methods [4], [24], [1], [27] in CrowdAI dataset.

Table 3: Experimental results of polygon extraction methods on the Strutured3D validation set.

Method	Room			Corner		
	**Prec.↑**	**Rec.↑**	**F1↑**	**Prec.↑**	**Rec.↑**	**F1↑**
Floor-SP [28]	89.0	88.0	88.0	81.0	73.0	76.0
HAWP [29]	77.7	87.6	82.3	65.8	77.0	70.9
LETR [30]	94.5	90.0	92.2	79.7	78.2	78.9
MonteFloor [31]	95.6	94.4	95.0	88.5	77.2	82.5
HEAT [32]	96.9	94.0	95.4	81.7	83.2	82.5
RoomFomer [33]	97.9	96.7	97.3	**89.1**	85.3	**87.2**
UniVector	**98.5**	**97.1**	**97.8**	86.2	**85.5**	85.8

Additionally, we present representative visualization results. As illustrated in Fig. 3, UniVector achieves optimal results in both detection accuracy and shape fidelity. In contrast, both FFL [27] and HiSup [24] suffer from false positives and missed detections. This is due to the segmentation masks of objects that are sufficiently close to each other, leading to adhesion and merged vector structures. Although PolyR-CNN demonstrates competitive detection performance, its vectorization of complex shapes lacks precision, exhibiting redundant vertices and overly smooth contours. As demonstrated in Fig. 4, UniVector significantly reduces the global impact of erroneous nodes compared to RoomFormer, achieving superior visual accuracy in structural representation.

Polyline Extraction. We conduct qualitative comparisons between

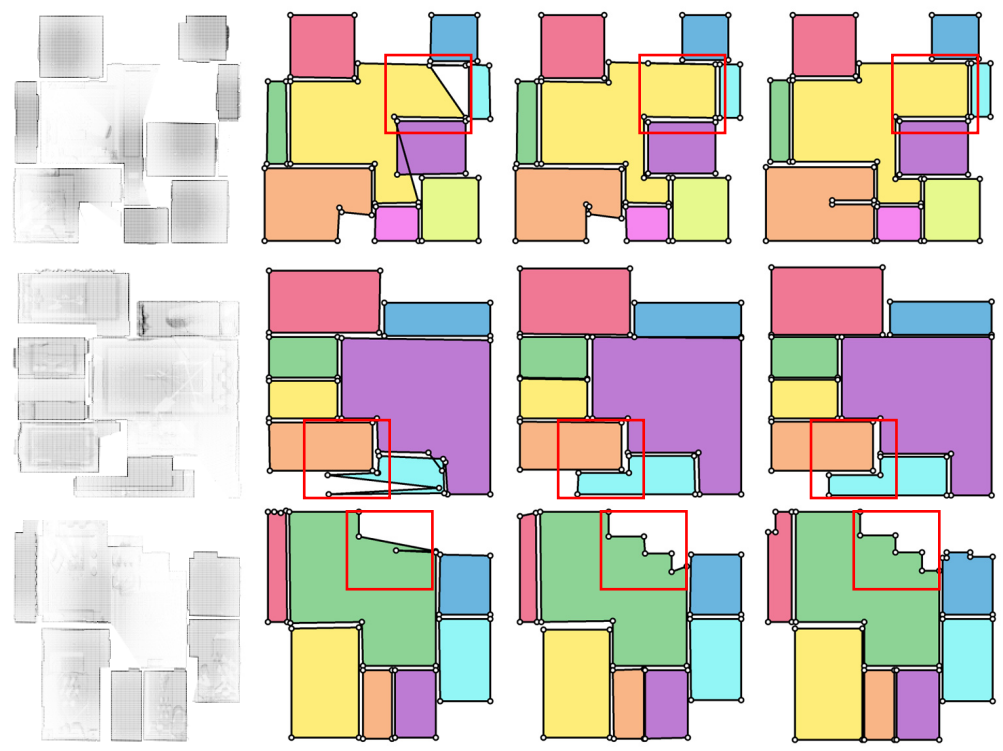

Figure 4: Qualitative comparison with other room extraction method [33] in Strutured3D dataset.

UniVector and competing methods, as shown in Fig. 5. UniVector provides best visual fidelity, smoothly delineating road boundaries without discernible topological errors. In contrast, Deeproadmapper [35] exhibits the poorest visual performance, with noticeable disconnections and bending. While VecRoad [34], Enhanced-iCurb [14], and RNGDet++ [25] show improved connectivity over Deeproadmapper, their corner representations remain geometrically crude, with abrupt angular transitions and residual topological inaccuracies.

Line Segment Extraction. We present qualitative comparison results in Fig. 6. The visualizations clearly show that Transformer-based methods (LETR and UniVector) significantly outperform L-CNN [37] and HAWP [29], effectively mitigating false line segment detections. Furthermore, UniVector reduces the noisy segments observed in LETR. These visual comparisons further validate the superior performance of UniVector.

1.5. Ablation Studies

In this section, we present more detailed ablation studies, including comparisons of vector encoding methods, different strategies for instance–geometry interaction, and the settings of hyperparameters.

1.5.1. Discussion of Unified Vector Encoding

Comparison of Different Encoding Methods. We compare different structured query encoding methods: random encoding [38], hierarchical

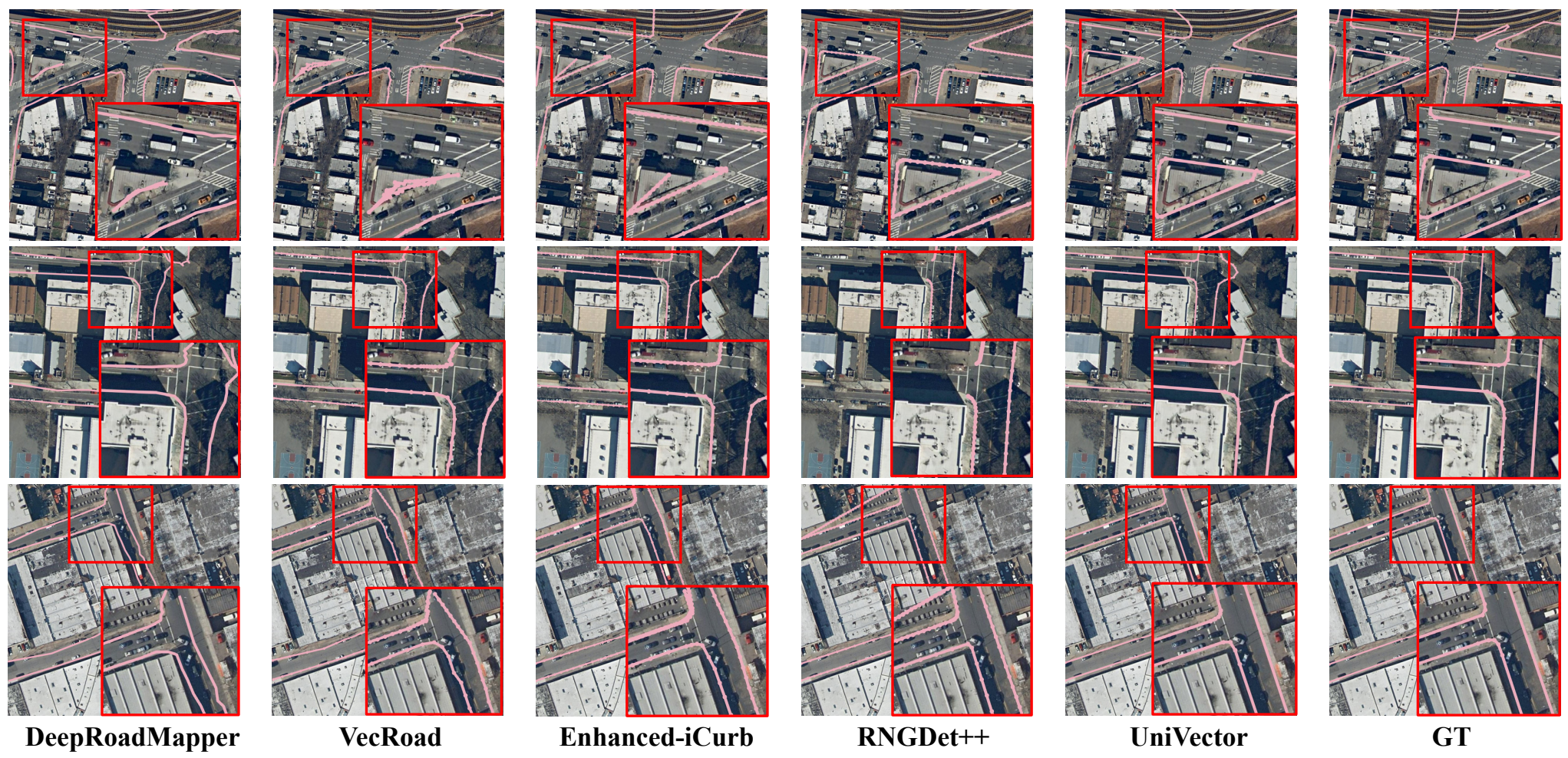

Figure 5: Qualitative comparison of road boundary extraction methods [14], [34], [25], [35] on the Topo-Boundary dataset.

encoding [23], and our proposed UVE, as shown in Fig. 7. Random encoding methods produce vectors with highly disordered shapes, failing to provide effective positional or geometric priors (Fig. 7(a)). While hierarchical encoding improves the quality of initialized vectors, it focuses solely on positional cues and struggles to capture geometric structures (Fig. 7(b)). In contrast, UVE holistically integrates instance detection and geometric deformation to encode instance-level and geometry-aware features (Fig. 7(c), enabling more effective initialization across diverse objects.

Parameters for Structured Queries. In Table 5, we investigate the impact of the number of structured queries, N, and the number of points, M, on performance and computational cost. Based on statistical analysis of dataset labels, we set the experimental ranges for N from 30 to 100 and M from 30 to 60. The results indicate that both performance and computational load increase with increasing N, peak at $N = 50$, and subsequently decline. For parameter M, we observe that the performance gap remains minimal across $M = 30, 40, 50$, therefore providing considerable flexibility in parameter selection. In this paper, we select $M = 40$.

1.5.2. Discussion of Instance-Geometry Interaction

What Have Structured Queries Learned? To better understand the capabilities of structured queries and the effect of instance-geometry in-

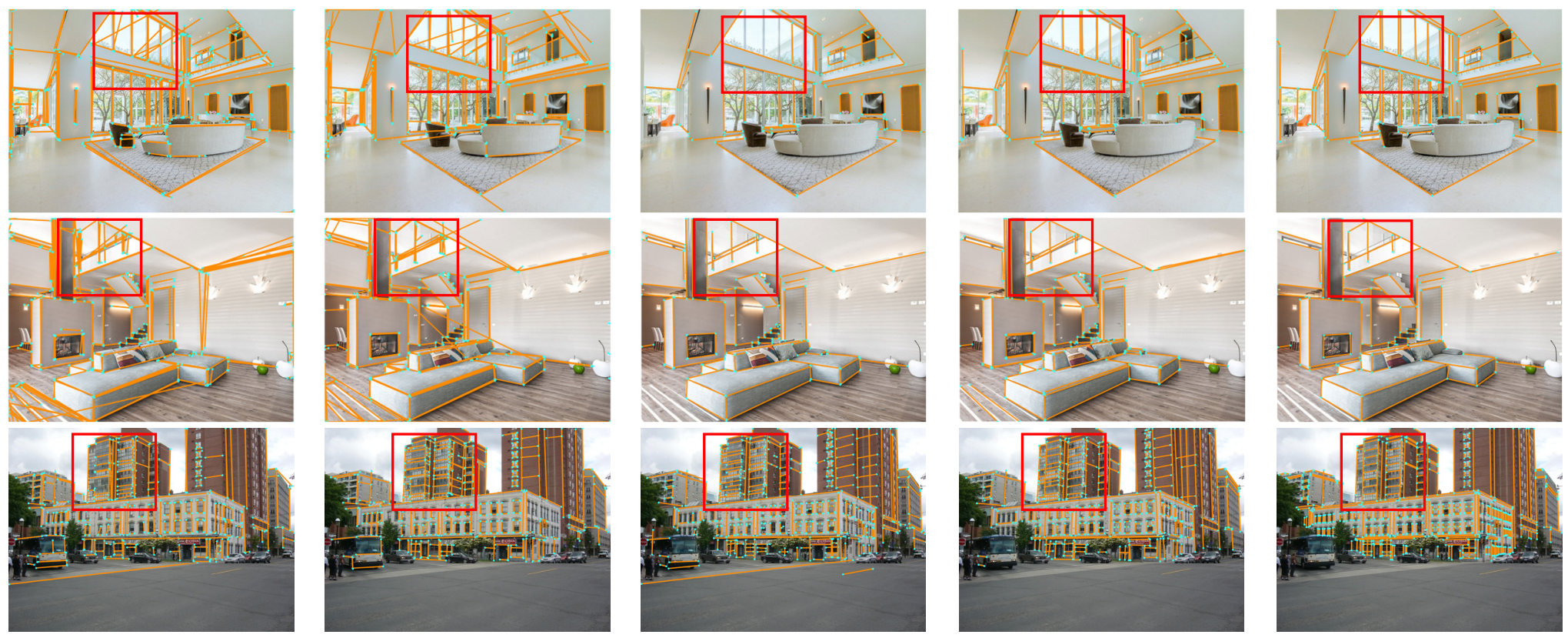

Figure 6: Qualitative comparison with other wireframe extraction methods [30], [36], [37] in Wireframe and York Urban datasets. The first two rows are from the Wireframe dataset, and the last row is from the York Urban dataset.

Table 4: Ablation studies on the three modules of UniVector across datasets Multi-Vector, CroadAI, Topo-Boundary, and Wireframe.

Baseline	IGID	UVE	DSC	Multi-Vector			CrowdAI	Topo-Boundary	Wireframe
				Building	Road Boundary	Center Line			
✓				39.6	77.2	78.3	63.9	78.8	62.3
✓	✓			45.2 (+5.6)	83.2 (+6.0)	83.8 (+5.5)	69.3 (+5.4)	85.6 (+6.8)	66.8 (+4.5)
✓	✓	✓		47.6 (+2.4)	85.4 (+2.2)	86.3 (+2.5)	71.5 (+2.2)	87.5 (+1.9)	68.2 (+1.4)
✓	✓	✓	✓	**49.4 (+1.8)**	**87.8 (+2.4)**	**88.6 (+2.3)**	**72.8 (+1.3)**	**90.3 (+2.8)**	**69.1 (+0.9)**

teraction, we visualize the attention maps across decoder layers, as shown in Fig. 8. These attention maps highlight the relationships between reference points and their corresponding sampling points. The visualizations reveal that instance queries primarily focus on capturing global vector information, while geometry queries emphasize local structural details. Furthermore, across different decoder layers, instance-level and geometry-level reference points undergo iterative cross-level interactions, progressively refining their positions toward optimal locations. The dynamic refinement

Table 5: Ablation on N and M in Structured Queries.

Query Num.	Point Num.	Building	Road Boundary	Center Line	GPU Mem.
30	40	44.5	82.1	84.1	2305 M
40	40	48.6	85.1	84.1	2898 M
**50**	40	**50.8**	**85.4**	**85.1**	3648 M
60	40	43.5	79.8	81.3	4525 M
50	30	**49.9**	87.6	88.5	2756 M
50	**40**	49.4	87.8	**88.6**	3648 M
50	50	49.8	**88.4**	87.8	4751 M
50	60	47.5	84.3	86.5	6071 M

Table 6: Ablation on different interaction strategies. The G-G, I-I, and I-G denote geometry-level, instance-level, and instance-to-geometry query interactions, respectively.

Interaction	Multi-Vector			GPU Mem.
	Building	Road Boundary	Center Line	
Full	44.6	82.5	85.3	5092 M
G-G	43.2	83.1	83.4	3648 M
G-G + I-G	48.6 (+5.4)	87.1 (+4.0)	87.5 (+4.1)	3648 M
**G-G + I-G + I-I (Ours)**	**49.4 (+0.8)**	**87.8 (+0.7)**	**88.6 (+1.1)**	3648 M

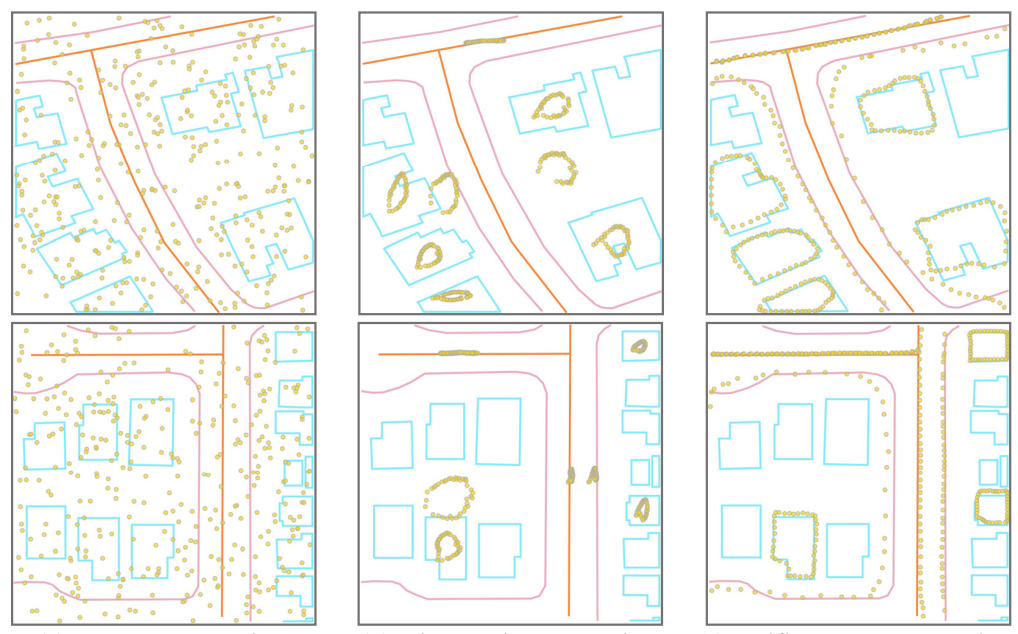

Figure 7: Visualization of different query encoding methods.

enabled by instance-geometry interaction significantly enhances vector extraction precision, demonstrating how structured queries effectively leverage both global and local features to boost model performance.

How to Implement Instance-Geometry Interaction? In Table 6, we analyze the impact of different interaction learning strategies, where G-G, I-I, and I-G denote geometry-level, instance-level, and instance-to-geometry query interactions, respectively. It should be noted that the G-G and I-G mentioned here specifically refer to geometric-layer and instance-geometry interaction within the same instance. The Full denotes the global interaction of intra-instance and inter-instance queries as described in [33]. The experimental results demonstrate that both global interaction (Full) and geometry-level interaction (G-G) yield suboptimal performance, whereas introducing instance-geometry interaction (I-G) and instance-level interaction

Table 7: Ablation on loss components in dynamic shape constraint.

Loss	DM	$\mathcal{L}_{kp}$	$\mathcal{L}_{dir}$	Multi-Vector		
				Building	Road Boundary	Center Line
Smooth l_1	—	—	—	47.6	85.4	86.3
	✓	✓		48.8 (+1.2)	86.7 (+1.3)	87.4 (+1.1)
DSC	✓		✓	47.3 (-0.3)	84.9 (-0.5)	85.6 (-0.7)
	✓	✓	✓	**49.4 (+1.8)**	**87.8 (+2.4)**	**88.6 (+2.3)**

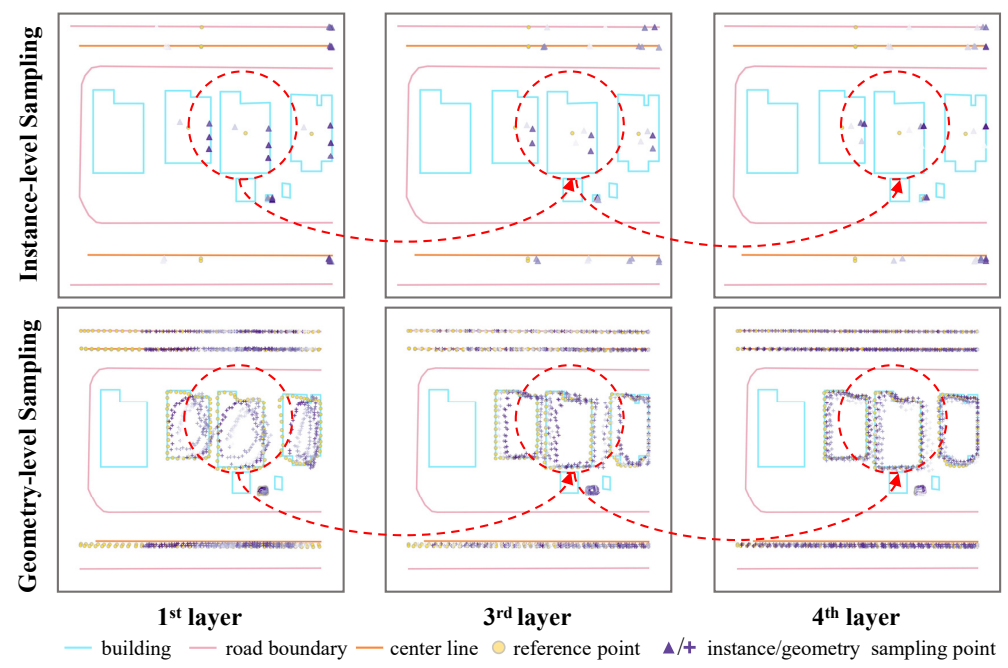

Figure 8: Attention maps of structured queries at different layers.

Table 8: Ablation on loss factors of L_{kp} and L_{dir}.

$\mathcal{L}_{kp}$: $\mathcal{L}_{dir}$	Multi-Vector		
	Building	Road Boundary	Center Line
1:1	47.5	85.4	85.9
5:1	48.6	86.3	86.3
**10:1**	**49.4**	**87.8**	**88.6**
5:1	47.6	85.3	86
10:1	45.2	83.9	84.2

(I-I) significantly enhances model accuracy. Notably, while global interaction (Full) outperforms geometry-level interaction (G-G), it incurs 40% higher computational overhead. This limitation likely stems from interference between points of different instances during cross-instance interactions. In contrast, instance-geometry interaction (I-G) and instance-level interaction (I-I) achieve substantial performance gains with negligible computational overhead, effectively balancing efficiency and precision.

1.5.3. Discussion of Dynamic Shape Constraint

We conduct ablation experiments on the loss functions and loss weights within the dynamic shape constraints. As shown in Table 7, when removing the dynamic shape constraints and using only a simple smooth l_1 loss, the model performance drops by 1.8%, 2.4%, and 2.3% for buildings, road boundaries, and center lines, respectively. Furthermore, employing only the directional loss within DCS results in performance degradation, demonstrating that the directional loss fails to handle structurally similar yet positionally parallel configurations. The combined use of keypoint loss L_{kp} and directional loss L_{dir} achieves the best results. Table 8 demonstrates that the optimal loss weight ratio between them is 10:1.

References

[1] W. Jiao, C. Persello, G. Vosselman, PolyR-CNN: R-CNN for end-to-end polygonal building outline extraction, ISPRS J. Photogramm. Remote Sens. 218 (2024) 33–43. doi:10.1016/j.isprsjprs.2024.10.006.

[2] S. Zorzi, F. Fraundorfer, Re: Polyworld-a graph neural network for polygonal scene parsing, in: Proceedings of the IEEE/CVF International Conference on Computer Vision, 2023, pp. 16762–16771.

[3] L. Wang, M. Dai, J. He, J. Huang, Regularized Primitive Graph Learning for Unified Vector Mapping, in: Proceedings of the IEEE/CVF International Conference on Computer Vision, 2023, pp. 16817–16826.

[4] S. Zorzi, S. Bazrafkan, S. Habenschuss, F. Fraundorfer, PolyWorld: Polygonal Building Extraction with Graph Neural Networks in Satellite Images, in: IEEE/CVF Conf. Comput. Vis. Pattern Recognit. (CVPR), IEEE, New Orleans, LA, USA, 2022, pp. 1938–1947. doi:10.1109/CVPR52688.2022.00189.

[5] J. Shin, H. Jeong, F. Rameau, D. Kum, Instagram: Instance-level graph modeling for vectorized hd map learning, IEEE Transactions on Intelligent Transportation Systems (2025).

[6] S. P. Mohanty, Crowdai mapping challenge 2018 : Baseline with mask rcnn, https://github.com/crowdai/crowdai-mapping-challenge-mask-rcnn, 2018.

[7] L. Chen, C. Sima, Y. Li, Z. Zheng, J. Xu, X. Geng, H. Li, C. He, J. Shi, Y. Qiao, et al., Persformer: 3d lane detection via perspective transformer and the openlane benchmark, in: European Conference on Computer Vision, Springer, 2022, pp. 550–567.

[8] Z. Li, J. D. Wegner, A. Lucchi, Topological map extraction from overhead images, in: Proceedings of the IEEE/CVF International Conference on Computer Vision, 2019, pp. 1715–1724.

[9] T.-Y. Lin, M. Maire, S. Belongie, J. Hays, P. Perona, D. Ramanan, P. Dollár, C. L. Zitnick, Microsoft coco: Common objects in context, in: Computer vision–ECCV 2014: 13th European conference, zurich,

Switzerland, September 6-12, 2014, proceedings, part v 13, Springer, 2014, pp. 740–755.

[10] E. Maggiori, Y. Tarabalka, G. Charpiat, P. Alliez, Can semantic labeling methods generalize to any city? the inria aerial image labeling benchmark, in: 2017 IEEE International geoscience and remote sensing symposium (IGARSS), IEEE, 2017, pp. 3226–3229.

[11] J. Zheng, J. Zhang, J. Li, R. Tang, S. Gao, Z. Zhou, Structured3d: A large photo-realistic dataset for structured 3d modeling, in: Computer Vision–ECCV 2020: 16th European Conference, Glasgow, UK, August 23–28, 2020, Proceedings, Part IX 16, Springer, 2020, pp. 519–535.

[12] A. Avetisyan, T. Khanova, C. Choy, D. Dash, A. Dai, M. Nießner, Scenecad: Predicting object alignments and layouts in rgb-d scans, in: Computer Vision–ECCV 2020: 16th European Conference, Glasgow, UK, August 23–28, 2020, Proceedings, Part XXII 16, Springer, 2020, pp. 596–612.

[13] B. Hariharan, P. Arbeláez, L. Bourdev, S. Maji, J. Malik, Semantic contours from inverse detectors, in: 2011 international conference on computer vision, IEEE, 2011, pp. 991–998.

[14] Z. Xu, Y. Sun, M. Liu, Topo-Boundary: A Benchmark Dataset on Topological Road-Boundary Detection Using Aerial Images for Autonomous Driving, IEEE Robotics and Automation Letters 6 (2021) 7248–7255. doi:10.1109/LRA.2021.3097512.

[15] D. Neven, B. De Brabandere, S. Georgoulis, M. Proesmans, L. Van Gool, Towards end-to-end lane detection: an instance segmentation approach, in: 2018 IEEE intelligent vehicles symposium (IV), IEEE, 2018, pp. 286–291.

[16] X. Pan, J. Shi, P. Luo, X. Wang, X. Tang, Spatial as deep: Spatial cnn for traffic scene understanding, in: Proceedings of the AAAI conference on artificial intelligence, volume 32, 2018.

[17] K. Behrendt, R. Soussan, Unsupervised labeled lane markers using maps, in: Proceedings of the IEEE/CVF international conference on computer vision workshops, 2019, pp. 0–0.

[18] K. Huang, Y. Wang, Z. Zhou, T. Ding, S. Gao, Y. Ma, Learning to parse wireframes in images of man-made environments, in: Proceedings of the IEEE Conference on Computer Vision and Pattern Recognition, 2018, pp. 626–635.

[19] P. Denis, J. H. Elder, F. J. Estrada, Efficient edge-based methods for estimating manhattan frames in urban imagery, in: Computer Vision–ECCV 2008: 10th European Conference on Computer Vision, Marseille, France, October 12-18, 2008, Proceedings, Part II 10, Springer, 2008, pp. 197–210.

[20] J.-T. Lee, H.-U. Kim, C. Lee, C.-S. Kim, Semantic line detection and its applications, in: Proceedings of the IEEE International Conference on Computer Vision, 2017, pp. 3229–3237.

[21] K. He, X. Zhang, S. Ren, J. Sun, Deep residual learning for image recognition, in: Proceedings of the IEEE conference on computer vision and pattern recognition, 2016, pp. 770–778.

[22] H. Zhang, F. Li, S. Liu, L. Zhang, H. Su, J. Zhu, L. M. Ni, H.-Y. Shum, Dino: Detr with improved denoising anchor boxes for end-to-end object detection, arXiv preprint arXiv:2203.03605 (2022).

[23] B. Liao, S. Chen, Y. Zhang, B. Jiang, Q. Zhang, W. Liu, C. Huang, X. Wang, Maptrv2: An end-to-end framework for online vectorized hd map construction, International Journal of Computer Vision (2024) 1–23.

[24] B. Xu, J. Xu, N. Xue, G.-S. Xia, Accurate polygonal mapping of buildings in satellite imagery, arXiv preprint arXiv:2208.00609 (2022).

[25] Z. Xu, Y. Liu, Y. Sun, M. Liu, L. Wang, Rngdet++: Road network graph detection by transformer with instance segmentation and multi-scale features enhancement, IEEE Robotics and Automation Letters 8 (2023) 2991–2998.

[26] C. Hetang, H. Xue, C. Le, T. Yue, W. Wang, Y. He, Segment anything model for road network graph extraction, in: Proceedings of the IEEE/CVF Conference on Computer Vision and Pattern Recognition, 2024, pp. 2556–2566.

[27] N. Girard, D. Smirnov, J. Solomon, Y. Tarabalka, Polygonal building extraction by frame field learning, in: Proceedings of the IEEE/CVF Conference on Computer Vision and Pattern Recognition, 2021, pp. 5891–5900.

[28] J. Chen, C. Liu, J. Wu, Y. Furukawa, Floor-sp: Inverse cad for floorplans by sequential room-wise shortest path, in: Proceedings of the IEEE/CVF International Conference on Computer Vision, 2019, pp. 2661–2670.

[29] N. Xue, T. Wu, S. Bai, F. Wang, G.-S. Xia, L. Zhang, P. H. Torr, Holistically-attracted wireframe parsing, in: Proceedings of the IEEE/CVF Conference on Computer Vision and Pattern Recognition, 2020, pp. 2788–2797.

[30] Y. Xu, W. Xu, D. Cheung, Z. Tu, Line segment detection using transformers without edges, in: Proceedings of the IEEE/CVF Conference on Computer Vision and Pattern Recognition, 2021, pp. 4257–4266.

[31] S. Stekovic, M. Rad, F. Fraundorfer, V. Lepetit, Montefloor: Extending mcts for reconstructing accurate large-scale floor plans, in: Proceedings of the IEEE/CVF International Conference on Computer Vision, 2021, pp. 16034–16043.

[32] J. Chen, Y. Qian, Y. Furukawa, HEAT: Holistic Edge Attention Transformer for Structured Reconstruction, in: Proceedings of the IEEE/CVF Conference on Computer Vision and Pattern Recognition, 2022, pp. 3866–3875.

[33] Y. Yue, T. Kontogianni, K. Schindler, F. Engelmann, Connecting the dots: Floorplan reconstruction using two-level queries, in: Proceedings of the IEEE/CVF Conference on Computer Vision and Pattern Recognition, 2023, pp. 845–854.

[34] Y.-Q. Tan, S.-H. Gao, X.-Y. Li, M.-M. Cheng, B. Ren, Vecroad: Point-based iterative graph exploration for road graphs extraction, in: Proceedings of the IEEE/CVF Conference on Computer Vision and Pattern Recognition, 2020, pp. 8910–8918.

[35] G. Máttyus, W. Luo, R. Urtasun, Deeproadmapper: Extracting road topology from aerial images, in: Proceedings of the IEEE international conference on computer vision, 2017, pp. 3438–3446.

[36] N. Xue, T. Wu, S. Bai, F.-D. Wang, G.-S. Xia, L. Zhang, P. H. Torr, Holistically-attracted wireframe parsing: From supervised to self-supervised learning, IEEE Transactions on Pattern Analysis and Machine Intelligence (2023).

[37] Y. Zhou, H. Qi, Y. Ma, End-to-end wireframe parsing, in: Proceedings of the IEEE/CVF International Conference on Computer Vision, 2019, pp. 962–971.

[38] N. Carion, F. Massa, G. Synnaeve, N. Usunier, A. Kirillov, S. Zagoruyko, End-to-end object detection with transformers, in: European conference on computer vision, Springer, 2020, pp. 213–229.
